# Contribution of local amyloid-β and tau burden to hypometabolism in autosomal-dominant Alzheimer’s disease

**DOI:** 10.1093/braincomms/fcaf508

**Published:** 2026-01-02

**Authors:** Catarina Tristão-Pereira, Stephanie Langella, Ana Baena, Natalia Londono, Justin S Sanchez, Lusiana Martinez, Sergio Alvarez, Monica Vidal, David Aguillon, Yi Su, Hillary Protas, Michael J Properzi, Vincent Malotaux, Bing He, Averi Giudicessi, Eric M Reiman, Bernard J Hanseeuw, Yakeel T Quiroz

**Affiliations:** Department of Psychiatry, Massachusetts General Hospital, Harvard Medical School, Boston, MA 02114, USA; Department of Psychological and Brain Sciences, Boston University, Boston, MA 02215, USA; Department of Psychiatry, Massachusetts General Hospital, Harvard Medical School, Boston, MA 02114, USA; Grupo de Neurociencias de Antioquia, Universidad de Antioquia, Medellín 050010, Colombia; Grupo de Neurociencias de Antioquia, Universidad de Antioquia, Medellín 050010, Colombia; Department of Psychiatry, Massachusetts General Hospital, Harvard Medical School, Boston, MA 02114, USA; Department of Psychiatry, Massachusetts General Hospital, Harvard Medical School, Boston, MA 02114, USA; Department of Psychological and Brain Sciences, Boston University, Boston, MA 02215, USA; Hospital Pablo Tobón Uribe, Medellín 050034, Colombia; Hospital Pablo Tobón Uribe, Medellín 050034, Colombia; Grupo de Neurociencias de Antioquia, Universidad de Antioquia, Medellín 050010, Colombia; Banner Alzheimer’s Institute and Arizona Alzheimer’s Consortium, Phoenix, AZ 85006, USA; Banner Alzheimer’s Institute and Arizona Alzheimer’s Consortium, Phoenix, AZ 85006, USA; Department of Radiology, Massachusetts General Hospital, Harvard Medical School, Boston, MA 02129, USA; Department of Psychiatry, Massachusetts General Hospital, Harvard Medical School, Boston, MA 02114, USA; Department of Psychological and Brain Sciences, Boston University, Boston, MA 02215, USA; Department of Psychiatry, Massachusetts General Hospital, Harvard Medical School, Boston, MA 02114, USA; Department of Psychological and Brain Sciences, Boston University, Boston, MA 02215, USA; Department of Psychiatry, Massachusetts General Hospital, Harvard Medical School, Boston, MA 02114, USA; Department of Psychological and Brain Sciences, Boston University, Boston, MA 02215, USA; Banner Alzheimer’s Institute and Arizona Alzheimer’s Consortium, Phoenix, AZ 85006, USA; Department of Psychiatry, Massachusetts General Hospital, Harvard Medical School, Boston, MA 02114, USA; Université Catholique de Louvain, Brussels 1348, Belgium; Department of Psychiatry, Massachusetts General Hospital, Harvard Medical School, Boston, MA 02114, USA; Department of Psychological and Brain Sciences, Boston University, Boston, MA 02215, USA; Grupo de Neurociencias de Antioquia, Universidad de Antioquia, Medellín 050010, Colombia

**Keywords:** FDG–PET, cerebral hypometabolism, autosomal-dominant Alzheimer’s disease, imaging biomarkers, cognition

## Abstract

Glucose hypometabolism is observed in early Alzheimer’s disease. However, there are regional discrepancies in hypometabolism and Alzheimer’s pathological markers. We examined the local and global contributions of amyloid-β and tau pathology to glucose metabolism and their interplay in memory decline in Presenilin-1 E280A mutation carriers and non-carriers from the largest autosomal-dominant Alzheimer’s disease kindred. This cross-sectional study included 43 mutation carriers (6 cognitively impaired) and 39 non-carriers from the Colombia-Boston Biomarker Study. Glucose metabolism was assessed with [18F]fluorodeoxyglucose PET, and memory performance with the Consortium to Establish a Registry for Alzheimer’s Disease word list learning. A subgroup of 22 carriers and 26 non-carriers additionally had measures of amyloid-β and tau using 11C-Pittsburgh compound B and 18F-flortaucipir PET, respectively. First, we compared regional glucose metabolism between groups using the Wilcoxon rank-sum test. Then, we studied regional glucose metabolism associations with age, co-localized amyloid-β and tau pathology, and memory using Spearman correlation. Local specificity was assessed by partial correlations controlling for global amyloid-β and tau burden. Finally, we studied whether the link between Alzheimer’s pathology and memory was mediated by regional glucose hypometabolism. Mutation carriers exhibited lower glucose metabolism in the precuneus and isthmus cingulate compared to non-carriers. Hypometabolism correlated locally with greater tau accumulation in the medial temporal lobe, inferior temporal gyrus and prefrontal cortex, and with greater amyloid-β accumulation in the inferior temporal gyrus in carriers. These associations were no longer significant when controlled for global pathology, except for the frontal tau-hypometabolism correlation, which was independent of global tau burden, suggesting local specificity. Additionally, lower memory performance in carriers was associated with hypometabolism in regions typically affected by tau. The mediation analysis revealed a region-specific interplay in pathology, with the associations of amyloid-β and tau pathology with memory decline being mediated by hypometabolism in the inferior temporal. Our findings highlight the metabolic vulnerability of the precuneus in early stages, supporting a common pathophysiology between autosomal-dominant and sporadic Alzheimer’s disease. The lack of local correlations between amyloid-β, tau and hypometabolism suggests that distant effects may explain the regional discrepancies between pathology accumulation and metabolic alterations. This study describes a model where pathology advances and interacts in a region-specific manner to impact clinical outcomes, underscoring the importance of regional [18F]fluorodeoxyglucose PET as an independent predictor of cognitive decline. Overall, our findings improve understanding of the spatial progression of pathology, which could have important implications in disease management.

## Teaser

Tristão-Pereira *et al*. investigate the local and global contributions of amyloid-β and tau to glucose metabolism in autosomal-dominant Alzheimer’s disease using PET. Their findings suggest that distant rather than local effects may explain regional discrepancies between pathology accumulation and metabolic decline, underscoring that region-specific pathology interactions contribute to memory function.

## Graphical Abstract

**Graphical Abstract:**
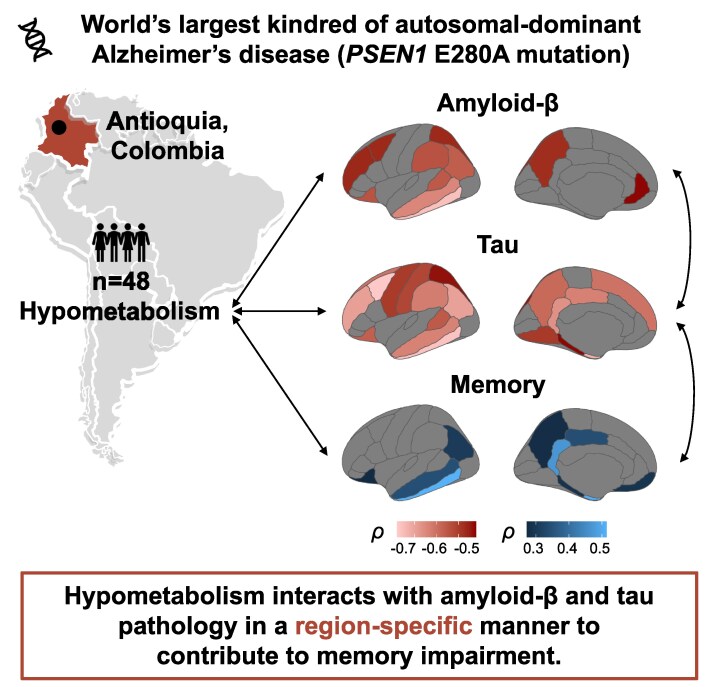

## Introduction

Cerebral glucose hypometabolism, measured with [18F]fluorodeoxyglucose (FDG) PET, is a primary feature of Alzheimer’s disease (AD).^1^ Hypometabolism occurs slowly with normal aging^2-4^ and accelerates along AD progression, typically affecting temporal and parietal brain regions in mild cognitive impairment (MCI),^5,6^ extending to the frontal lobe in sporadic AD.^7-9^ In autosomal-dominant AD (ADAD), a genetically determined form of early-onset AD, hypometabolism begins in the precuneus approximately a decade prior to the estimated onset of clinical symptoms.^10-12^

Within the Amyloid, Tau and Neurodegeneration research framework, hypometabolism follows the accumulation of amyloid-β (Aβ) plaques and tau tangles and precedes neuronal loss.^1^ The presence of Aβ is believed to promote tau spread from the medial temporal lobe to neocortical regions.^13^ Widespread tau then becomes closely linked to neurodegeneration, resulting in cognitive decline.^14^ However, the spatio-temporal interplay between Aβ, tau and hypometabolism in the disease progression is unclear. Some studies have not found any associations between glucose metabolism and Aβ pathology in sporadic AD^15-17^ or ADAD,^18^ while others have shown that hypometabolism and Aβ accumulation are locally correlated in temporal and parietal brain regions.^19-21^ The local specificity of these correlations has been questioned as the pattern of hypometabolism might depend on cortex-wide Aβ accumulation rather than regional deposits of fibrillar Aβ plaques.^22^ In turn, regional correlations between hypometabolism and tau in the entorhinal and inferior temporal cortices have been more consistently reported in sporadic AD^23-25^ and ADAD,^18,26^ though their local specificity has not been investigated. Given that the FDG-PET signal reflects, in part, neuronal loss, hypometabolism might be more influenced by intraneuronal tau tangles than extracellular Aβ.^27^

Thus, several studies have sought to determine whether glucose metabolism offers insights into cognitive function above the influence of AD pathology. Hypometabolism predicts cognitive decline from normal aging^6,23,28^ and is associated with memory deterioration in AD.^29-31^ Consistent with a model where tau and hypometabolism drive neurodegeneration before clinical symptoms, Aβ accumulation alone does not explain cognitive impairment in AD.^15,32,33^ Yet, conflicting findings pertaining to the role of tau in these associations cast doubt on whether cognitive correlates of hypometabolism are independent of^34^ or mediated by tau pathology.^35^

Together, existing literature improves understanding of the role glucose hypometabolism plays in the AD pathological cascade. However, there is inconsistent evidence for the contribution of regional Aβ and tau pathology to AD-related hypometabolism and their interplay in downstream cognitive deterioration. To investigate this, we propose to study FDG-PET data in *Presenilin-1* (*PSEN1*) E280A mutation carriers from the largest ADAD Colombian kindred. In this kindred, carriers develop MCI at a median age of 44 years and AD dementia at 49 years.^36^ Despite showing a more rapid progression,^37^ ADAD can serve as a roadmap for the late-onset form of the disease, given its well-characterized trajectory. Here, our first aim is to determine the regional pattern of glucose metabolism across the AD continuum in *PSEN1* mutation carriers and its age-related changes. Second, we aim to study cortical and subcortical glucose metabolism in relation to co-localized Aβ and tau burden, while accounting for global pathology burden. Third, we aim to examine the memory correlates of regional glucose metabolism and determine whether these are influenced by AD pathology. We hypothesize that tau, rather than Aβ, will primarily underlie FDG-PET reductions in brain regions of early pathology accumulation and that region-specific hypometabolism will correlate with cognitive impairment in ADAD.

## Materials and methods

### Study participants

In this study, we used data from *PSEN1* E280A carriers and age-matched non-carrier family members from the Colombia-Boston (COLBOS) Biomarker Study, conducted at the Massachusetts General Hospital (Boston, USA) in collaboration with the Neuroscience Group of Antioquia at the University of Antioquia and the Pablo Tobón Uribe Hospital (Medellin, Colombia).^36^ All participants with available FDG-PET scans and neuropsychological assessments were included in the study. Participants were excluded if they had a significant medical, psychiatric or neurological disorder or a history of stroke, seizures or substance abuse. A subset of participants also had [11C] Pittsburgh compound B (PiB) and [18F]flortaucipir (FTP) PET scans available for Aβ and tau quantification. All participants provided written informed consent before study assessments and were compensated. This study was approved by the local institutional review boards of the Massachusetts General Hospital and the University of Antioquia. Researchers and participants were blind to genetic status.

### Neuropsychological assessment

Neuropsychological evaluations were conducted in Spanish at the University of Antioquia within six months of neuroimaging data collection. The Functional Assessment Staging Tool (FAST; range: 1–7) was used to determine cognitive status, with a score ≤ 2 indicating no objective cognitive impairment.^38^ The Mini-Mental State Examination (MMSE; range: 0–30) was used to assess global cognitive function^39^ and the Consortium to Establish a Registry for Alzheimer’s Disease (CERAD) word list learning (range: 0–30) was used to assess memory performance,^40^ one of the earliest indicators of preclinical changes in *PSEN1* E280A carriers.^41^ Other tests included the CERAD’s Semantic Fluency test^42^ and the Trail Making Test Part A (TMTA) to assess processing speed.^43^ The neuropsychological battery is detailed in Supplementary material and has been previously reported.^36,41^

### Image acquisition and processing

Structural T1-weighted MRI scans were acquired on a Siemens 3 Tesla Tim Trio scanner (repetition time = 2300 ms, echo time = 2.95 ms, flip angle = 9 degrees, voxel size = 1.05 × 1.05 × 1.2 mm). Images were processed using Freesurfer v7.2.0 to identify white and pial surfaces and to segment cortical and subcortical regions of interest (ROIs) from the Desikan atlas.^44,45^ Visual inspection of the outputs was conducted, and manual edits were made when necessary to ensure accurate segmentation.

FDG-PET scans were acquired on a 64-section Siemens Biograph mCT PET/CT. Intravenous injection of 5 mCi of FDG was administered 30 min before a dynamic emission scan of 6 × 5-minute frames in fasting conditions (≥5 h). FDG-PET images were reconstructed with a computed tomographic attenuation correction and were processed using PET Unified Pipelines, which included scanner harmonization to 8 mm, motion correction and frame summation.^46^ Aβ and tau data were acquired on a Siemens Emission Computed Axial Tomography High Resolution + PET scanner (3D mode; 63 image planes; 15.2 cm axial field of view; 5.6 mm transaxial resolution; 2.4 mm slice interval). Aβ data were acquired with a 60-minute dynamic protocol immediately after a 8.5–15 mCi bolus injection of PiB (12 × 15-second + 57 × 60-second frames) and analysed by the Logan reference method.^47^ Tau data were acquired 80–100 min after a 9.0–11.0 mCi bolus injection of FTP (4 × 5-minute frames). PET and MRI images were co-registered with affine transformations, and PET data were sampled using Freesurfer-derived Desikan ROIs. Standardized uptake value ratio (SUVR) for FDG and FTP data and distribution volume ratio (DVR) for PiB data were estimated. We used the cerebellar grey matter as the reference region due to its low intraindividual variability in glucose metabolism and to ensure comparability across PET modalities.^48^ Each PET regional SUVR and DVR underwent partial volume correction using the regional spread function method.^49^ This method reduces atrophy-related bias by modelling a spill-over matrix at the regional level based on individual anatomical segmentations and the scanner’s point spread function.^50^ Left and right hemispheres were averaged for each FDG, FTP and PiB ROIs. Composite regions were used to account for global Aβ and tau pathology in early accumulation regions, with frontal, lateral temporal, parietal and retrosplenial PiB uptake averaged to represent global Aβ burden^51^ and precuneus, entorhinal and inferior temporal FTP uptake averaged to represent global tau burden, respectively.^52^

### Statistical analysis

Demographic characteristics were compared between *PSEN1* mutation carriers and non-carriers using two-tailed *t*-tests for continuous variables and χ^2^-tests for categorical variables. First, we compared FDG SUVR across all cortical and subcortical ROIs between carriers and non-carriers using the Wilcoxon rank-sum test. Paired Wilcoxon rank-sum tests compared FDG SUVR in the left and right hemispheres. Effect sizes of those differences were assessed using the rank-biserial correlation *r*. In the ROIs with the largest between-group differences, we assessed the correlation between FDG SUVR and age using Spearman correlation. The same approach was used to compare FTP SUVR and PiB DVR between groups. Second, we conducted Spearman correlations between the same-region FDG SUVR and FTP SUVR, as well as FDG SUVR and PiB DVR across all cortical and subcortical ROIs in *PSEN1* mutation carriers and non-carriers. To understand whether the observed significant effects were due to global pathology rather than local specificity, we performed partial correlations controlling for global FTP SUVR in local FDG–FTP correlations and for global PiB DVR in local FDG–PiB correlations. In addition, to explore the interplay between co-localized pathology burden, local FDG–FTP correlations were controlled for local PiB DVR and local FDG–PiB correlations for local FTP SUVR. Lastly, we assessed the link between regional FDG SUVR and memory (CERAD’s word list learning) in *PSEN1* mutation carriers and non-carriers using Spearman correlation. Secondary models assessed correlations with semantic fluency (CERAD’s semantic fluency) and processing speed (TMTA time) as well. All associations in *PSEN1* mutation carriers were further adjusted for MMSE (global cognition) using partial Spearman correlation. Mediation analyses were then performed in the imaging sub-sample to study whether the effects of Aβ and tau burden on memory were mediated by glucose metabolism. In mediation models, the independent variable was either PiB DVR or FTP SUVR, the dependent variable was memory (CERAD’s word list learning), and the mediator was FDG SUVR. These models were carried out in the four ROIs with the largest differences between groups and/or the strongest correlations (precuneus, caudal middle frontal, inferior temporal and hippocampus) and were adjusted for *PSEN1* mutation status. We used R’s *mediation* package with 1000 bootstrapping simulations.

We used Bonferroni correction for multiple comparisons in all regional analyses and bootstrapping methods to calculate confidence intervals. Statistical significance was set at α=0.05. All analyses were performed in R (v4.4.1).

## Results

### Sample characteristics

A total of 43 *PSEN1* E280A mutation carriers and 39 non-carriers were included in this study (Table 1). Mean age was 38.2 ± 7.5 years in carriers and 39.6 ± 6.7 years in non-carriers. Age, sex or education years did not differ between groups. Among mutation carriers, six were cognitively impaired (FAST > 2), while all non-carriers were cognitively intact (*P* < 0.001). MMSE scores (*P* < 0.001), CERAD’s word list learning (*P* = 0.005) and TMTA times (*P* = 0.023) were significantly lower in carriers compared to non-carriers. Of those, a subgroup of 22 *PSEN1* mutation carriers and 26 non-carriers had PiB-PET and FTP-PET data available in addition to FDG-PET data and neuropsychological assessments. The demographics of this subgroup are comparable to the main sample (Supplementary Table 1).

**Table 1:** Sample demographics

	*PSEN1* E280A carriers (*n* = 43)	Non-carriers (*n* = 39)	*P*-value
Age (years)	38.2 ± 7.5	39.6 ± 6.7	0.399
Education (years)	10.2 ± 3.9	11.5 ± 4.1	0.140
Sex (*n*, %)			0.744
Female	29 (67%)	24 (62%)	
Male	14 (33%)	15 (38%)	
FAST			**<0**.**001**
≤2	37 (86%)	39 (100%)	
>2	6 (14%)	0	
MMSE	27.3 ± 2.4	28.8 ± 1.0	**<0**.**001**
CERAD’s word list learning	18.0 ± 5.1	20.8 ± 3.8	**0**.**005**
CERAD’s semantic fluency	21.4 ± 5.1	21.2 ± 3.8	0.845
TMTA time	53.9 ± 37.3	39.7 ± 13.5	**0**.**023**

Demographics were compared between *PSEN1* mutation carriers (*n* = 43) and non-carriers (*n* = 39) using two-tailed *t*-tests for continuous variables and *χ*^2^-tests for categorical variables. Bold font represents statistical significance (*P* < 0.05).

Continuous variables are represented as mean ± standard deviation and categorical variables as *n* (%).

CERAD, Consortium to Establish a Registry for Alzheimer’s Disease; FAST, Functional Assessment Staging Tool; MMSE, Mini-Mental State Examination; TMTA,Trail Making Test Part A.

### Regional patterns of glucose hypometabolism in *PSEN1* E280A carriers

**Figure 1:**
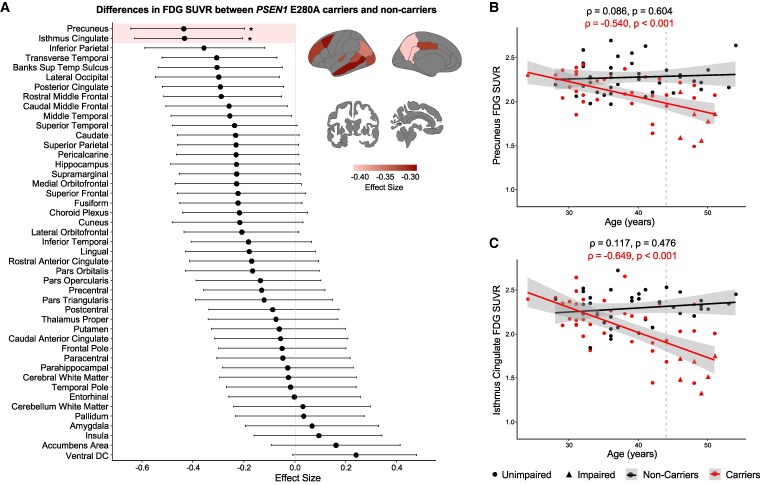
**Regional glucose metabolism differences between *PSEN1* mutation carriers and non-carriers.** Ordered effect sizes of the differences in regional FDG SUVR between groups calculated as the rank-biserial correlation r of the Wilcoxon rank-sum test (dots, *n* = 82) (**A**). Whiskers represent upper and lower bounds of bootstrap-derived 95% confidence intervals. Negative effect sizes indicate lower FDG SUVR in *PSEN1* mutation carriers compared to non-carriers. Brain projections highlight the regions with statistically significant differences between groups (*P* < 0.05), with light colours representing larger effect sizes. Regions highlighted within the forest plot represent models that survived Bonferroni correction for multiple comparisons. Scatter plots showing the associations between age and FDG SUVR in the precuneus (**B**) and isthmus cingulate (**C**) for *PSEN1* mutation carriers (*n* = 43) and non-carriers (*n* = 39), using Spearman correlation *ρ*. Data points represent subjects; colour lines represent estimated regression slopes; and shadows represent 95% confidence intervals. *: *p*_adj_ < 0.05. FDG, [18F]fluorodeoxyglucose; SUVR, standardized uptake value ratio; Banks Sup Temp Sulcus, banks of the superior temporal sulcus; Ventral DC, ventral diencephalon.

First, we assessed between-group differences in mean FDG uptake across bilateral cortical and subcortical regions. We observed a pattern of hypometabolism in parietal, temporal and frontal regions, with significantly lower glucose metabolism in *PSEN1* mutation carriers compared to non-carriers in the precuneus [*W* = 473, *r* = −0.436, 95% confidence interval (CI: −0.643, −0.186), *P* < 0.001, *p_adj_* = 0.027] and isthmus cingulate [*W* = 475, *r* = −0.434, 95% CI (−0.643, −0.219), *P* < 0.001, *p_adj_* = 0.027], after Bonferroni correction for multiple comparisons (Fig. 1A). We observed hemispheric differences in FDG SUVR, which were consistent in both carriers and non-carriers (Supplementary Fig. 1). In these brain regions, glucose metabolism was negatively correlated with age in carriers [precuneus: *ρ* = −0.540, 95% CI (−0.717, −0.282), *P* < 0.001; isthmus cingulate: *ρ* = −0.649, 95% CI (−0.788, −0.437), *P* < 0.001] but not in non-carriers [precuneus: *ρ* = 0.086, 95% CI (−0.257, 0.377), *P* = 0.604; isthmus cingulate: *ρ* = 0.117, 95% CI (−0.181, 0.399), *P* = 0.476] (Fig. 1BC). The intersection point was at ages 28.4 years in the precuneus and 31.6 years in the isthmus cingulate, which corresponds to 15.6 years and 12.4 years before the estimated age of clinical onset in carriers (44 years).^11^ The correlation between FDG uptake and age in carriers remained significant after adjusting for global cognition (Supplementary Table 2).

Additionally, regional differences in FTP SUVR revealed higher tau accumulation in *PSEN1* mutation carriers compared to non-carriers, mainly in medial temporal lobe structures, including the parahippocampal gyrus, amygdala, hippocampus and entorhinal cortex (Supplementary Fig. 2A). In contrast, regional PiB DVR showed widespread higher Aβ burden in carriers than non-carriers, with the greatest differences in basal ganglia and neocortical structures (Supplementary Fig. 2B).

### Local and global associations between hypometabolism and Aβ and tau pathology in *PSEN1* E280A carriers

**Figure 2:**
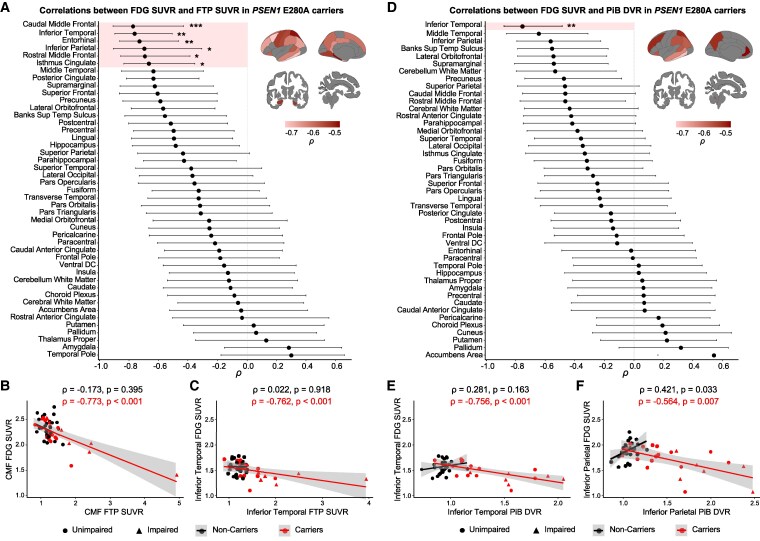
**Regional association between glucose metabolism and Aβ and tau pathology in *PSEN1* mutation carriers.** Ordered correlation coefficients *ρ* of the regional associations of FDG SUVR with FTP SUVR (**A**) and PiB DVR (**D**) in *PSEN1* mutation carriers (dots, *n* = 22) calculated from Spearman correlation. Whiskers represent upper and lower bounds of bootstrap-derived 95% confidence intervals. Brain projections highlight the regions with statistically significant correlations, with light colours representing larger effect sizes for negative correlations. Regions highlighted within the forest plot represent models that survived Bonferroni correction for multiple comparisons. Scatter plots showing the associations of FDG SUVR with FTP SUVR in the caudal middle frontal (**B**) and inferior temporal (**C**) and with PiB DVR in the inferior temporal (**E**) and inferior parietal (**F**) in *PSEN1* mutation carriers (*n* = 22) and non-carriers (*n* = 26), using Spearman correlation *ρ*. Data points represent subjects; colour lines represent estimated regression slopes; and shadows represent 95% confidence intervals. *: *p*_adj_ < 0.05, **: *p*_adj_ < 0.01, ***: *p*_adj_ < 0.001. DVR, distribution value ratio; FDG, [18F]fluorodeoxyglucose; FTP, [18F]flortaucipir; PiB, [11C]Pittsburgh compound **B**; SUVR, standardized uptake value ratio; Banks Sup Temp Sulcus, banks of the superior temporal sulcus; ventral DC, Ventral diencephalon; CMF, caudal middle frontal.

Further, we assessed the local associations of FDG SUVR with FTP SUVR and PiB DVR across cortical and subcortical regions in *PSEN1* mutation carriers and non-carriers. Glucose metabolism and tau accumulation were inversely associated in frontal, temporal and parietal regions in carriers (Fig. 2ABC). Specifically, glucose hypometabolism was locally correlated with greater tau burden after Bonferroni correction in the caudal middle frontal [*ρ* = −0.773, 95% CI (−0.909, −0.491), *P* < 0.001, *p_adj_* < 0.001], inferior temporal [*ρ* = −0.762, 95% CI (−0.905, −0.487), *P* < 0.001, *p_adj_* = 0.005], entorhinal [*ρ* = −0.730, 95% CI (−0.856, −0.476), *P* < 0.001, *p_adj_* = 0.009], inferior parietal [*ρ* = −0.696, 95% CI (−0.897, −0.326), *P* < 0.001, *p_adj_* = 0.023], rostral middle frontal [*ρ* = −0.693, 95% CI (−0.859, −0.383), *P* < 0.001, *p_adj_* = 0.023] and isthmus cingulate [*ρ* = −0.666, 95% CI (−0.832, −0.361), *P* = 0.001, *p_adj_* = 0.045]. To test for local specificity, we controlled local correlations between FDG SUVR and FTP SUVR in *PSEN1* mutation carriers for global tau pathology. Partial correlations remained significant in the caudal [*ρ* = −0.706, 95% CI (−0.869, −0.405), *P* < 0.001] and rostral [*ρ* = −0.473, 95% CI (−0.746, −0.064), *P* = 0.030] middle frontal, but not in the other brain regions (Table 2). Additionally, all these regional partial correlations remained unchanged when controlling for co-localized Aβ accumulation (Table 2).

**Table 2:** Local and global effects of Aβ and tau accumulation on glucose metabolism in *PSEN1* mutation carriers

Partial correlations between local FDG SUVR and FTP SUVR in *PSEN1* carriers			
Region	Simple correlation	Controlling for global FTP SUVR	Controlling for local PiB DVR
Caudal Middle Frontal	**−0.773** **[−0.908, −0.444]** **(*P*** **<** **0.001)**	**−0.706** **[−0.869, −0.405]** **(*P*** **<** **0.001)**	**−0.698** **[−0.865, −0.392]** **(*P*** **<** **0.001)**
Inferior Temporal	**−0.762** **[−0.894, −0.512]** **(*P*** **<** **0.001)**	−0.343 [−0.668, 0.092] (*P* = 0.128)	**−0.482** **[−0.751, −0.076]** **(*P*** **=** **0.027)**
Entorhinal	**−0.730** **[−0.854, −0.482]** **(*P*** **<** **0.001)**	−0.425 [−0.718, 0.000] (*P* = 0.055)	**−0.747** **[−0.889, −0.474]** **(*P*** **<** **0.001)**
Inferior Parietal	**−0.696** **[−0.894, −0.326]** **(*P*** **<** **0.001)**	−0.353 [−0.674, 0.080] (*P* = 0.116)	**−0.495** **[−0.758, −0.092]** **(*P*** **=** **0.023)**
Rostral Middle Frontal	**−0.693** **[−0.852, −0.361]** **(*P*** **<** **0.001)**	**−0.473** **[−0.746, −0.064]** **(*P*** **=** **0.030)**	**−0.584** **[−0.807, −0.215]** **(*P*** **=** **0.005)**
Isthmus Cingulate	**−0.666** **[−0.839, −0.337]** **(*P*** **=** **0.001)**	−0.157 [−0.283, 0.543] (*P* = 0.496)	**−0.635** **[−0.834, −0.292]** **(*P*** **=** **0.002)**
**Partial correlations between local FDG SUVR and PiB DVR in *PSEN1* carriers**			
** Region**	**Simple correlation**	**Controlling for global PiB DVR**	**Controlling for local FTP SUVR**
Inferior Temporal	**−0.756** **[−0.869, −0.498]** **(*P*** **<** **0.001)**	−0.228 [−0.481, 0.060] (*P* = 0.124)	**−0.465** **[−0.741, −0.054]** **(*P*** **=** **0.034)**

Local simple and partial correlations of FDG SUVR with FTP SUVR and PiB DVR in *PSEN1* mutation carriers (*n* = 22) in brain regions where simple correlations survived Bonferroni correction. Partial correlations were either controlled for global FTP SUVR or same-region PiB DVR and for global PiB DVR or same-region FTP SUVR. Spearman correlation coefficients *ρ*, bootstrap-derived 95% confidence intervals (CI), and *P*-values are shown for each correlation. Bold font represents statistical significance (*P* < 0.05). DVR, distribution value ratio; FDG, [18F]fluorodeoxyglucose; FTP, [18F]flortaucipir; PiB, [11C]Pittsburgh compound B; SUVR, standardized uptake value ratio.

The relationship between glucose metabolism and Aβ accumulation was not as extensive across the brain, with *PSEN1* mutation carriers showing a Bonferroni-corrected correlation between lower glucose metabolism and higher Aβ burden only in the inferior temporal [*ρ* = −0.756, 95% CI (−0.873, −0.500), *P* < 0.001, *p_adj_* = 0.005] (Fig. 2DEF). When controlling for global Aβ pathology, the partial correlation between FDG SUVR and PiB DVR in the inferior temporal was no longer significant [*ρ* = −0.228, 95% CI (−0.481, 0.060), *P* = 0.124; Table 2]. In contrast, this partial correlation remained unchanged when controlling for co-localized tau accumulation (Table 2). Same-region correlations between FDG SUVR and FTP SUVR as well as FDG SUVR and PiB DVR in carriers were independent of global cognition, with MMSE-adjusted correlations remaining significant (Supplementary Table 2).

In non-carriers, FDG SUVR did not correlate with either FTP SUVR or PiB DVR after Bonferroni correction (Supplementary Fig. 3). Local and partial correlations were similar in the whole imaging sub-sample as in *PSEN1* mutation carriers (Supplementary Table 3).

### Associations of regional hypometabolism with memory and their interplay with Aβ and tau pathology

**Figure 3:**
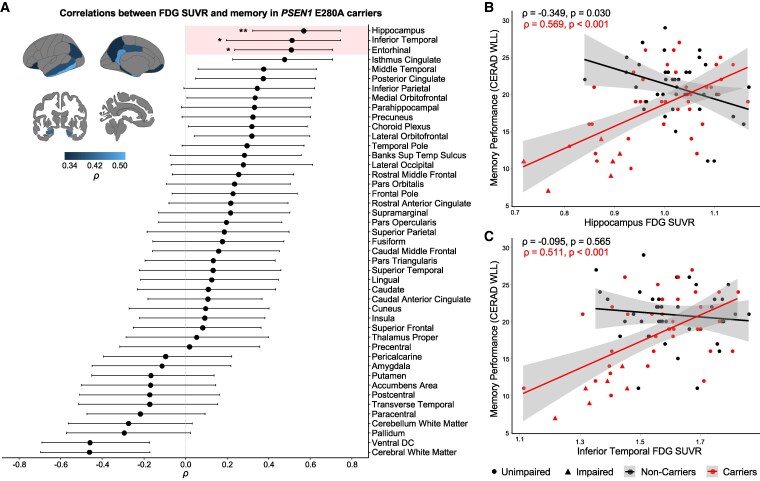
**Association between regional glucose metabolism and memory performance in *PSEN1* mutation carriers.** Ordered correlation coefficients *ρ* of the associations of regional FDG SUVR with CERAD word list learning (memory performance, (**A**) in *PSEN1* mutation carriers (dots, *n* = 43) calculated from Spearman correlation. Whiskers represent upper and lower bounds of bootstrap-derived 95% confidence intervals. Brain projections highlight the regions with statistically significant correlations, with light colours representing larger effect sizes for positive correlations. Regions highlighted within the forest plot represent models that survived Bonferroni correction for multiple comparisons. Scatter plots showing the associations of CERAD word list learning in the hippocampus (**B**) and inferior temporal (**C**) for *PSEN1* mutation carriers (*n* = 43) and non-carriers (*n* = 39), using Spearman correlation *ρ*. Data points represent subjects; colour lines represent estimated regression slopes; and shadows represent 95% confidence intervals. *: *p*_adj_ < 0.05, **: *p*_adj_ < 0.01. CERAD, Consortium to Establish a Registry for Alzheimer’s disease; FDG, [18F]fluorodeoxyglucose; SUVR , standardized uptake value ratio; Banks Sup Temp Sulcus, banks of the superior temporal sulcus; ventral DC, ventral diencephalon.

We then assessed the association between memory and glucose metabolism across cortical and subcortical brain regions. FDG uptake in temporal and parietal regions was correlated with the CERAD’s word list learning in *PSEN1* mutation carriers (Fig. 3A). We observed significant Bonferroni-corrected correlations between worse memory performance and hypometabolism in the hippocampus[*ρ* = 0.569, 95% CI (0.329, 0.745), *P* < 0.001, *p_adj_* = 0.005], inferior temporal [*ρ* = 0.511, 95% CI (0.202, 0.740), *P* < 0.001, *p_adj_* = 0.023] and entorhinal [*ρ* = 0.509, 95% CI (0.229, 0.708), *P* < 0.001, *p_adj_* = 0.023; Fig. 3BC]. Partial correlations between memory and FDG uptake remained statistically significant when adjusted for MMSE (Supplementary Table 2). In non-carriers, memory performance was not associated with glucose metabolism across the brain (Supplementary Fig. 4). Among other cognitive functions, reduced FDG uptake was associated with increased processing speed in carriers, in a pattern similar to that of memory, but not to semantic fluency (Supplementary Fig. 5).

**Figure 4:**
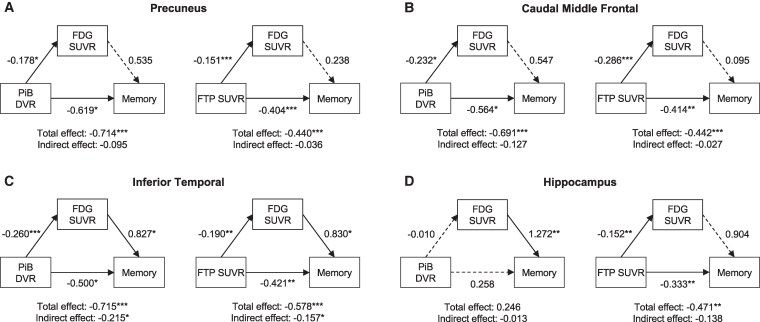
**Mediator role of glucose metabolism on the memory correlates of Aβ and tau pathology.** Associations of PiB DVR and FTP SUVR with memory performance (CERAD’s word list learning) mediated by FDG SUVR in the precuneus (**A**), caudal middle frontal (**B**), inferior temporal (**C**) and hippocampus (**D**) in the imaging sub-sample (*n* = 48). Mediation was performed with bootstrapping. Arrows indicate the effect of the exposure on the outcome. Solid/dashed lines represent statistically significant/non-significant associations (*P* < 0.05). The total and direct effects are the effects of the exposure on the outcome before and after accounting for the mediator, respectively; the indirect effect is the contribution of the mediator on the total effect; and the proportion mediated is the indirect effect over the total effect. Results are represented as standardized regression coefficients. *: *P* < 0.05, **: *P* < 0.01, ***: *P* < 0.001. DVR, distribution value ratio; FDG, [18F]fluorodeoxyglucose; FTP, [18F]flortaucipir; PiB, [11C]Pittsburgh compound **B**; SUVR, standardized uptake value ratio.

Lastly, we evaluated whether hypometabolism mediated the memory correlates of Aβ and tau pathology in relevant brain regions in the imaging sub-sample (*n* = 48) while adjusting for *PSEN1* mutation status. Region-specific mediations were observed. In the precuneus and caudal middle frontal, the effect of PiB DVR or FTP SUVR on memory was independent of FDG SUVR as the exposure-outcome pathway was statistically significant (total and direct effects) while the mediator-outcome pathway was not (indirect effect; Fig. 4AB). In the inferior temporal, FDG SUVR partially mediated the association between PiB DVR and memory [total effect: *β* = −0.715, 95% CI (−1.188, −0.280), *P* = 0.002; direct effect: *β* = −0.500, 95% CI (−0.961, −0.050), *P* = 0.022; indirect effect: *β* = −0.215, 95% CI (−0.487, −0.020), *P* = 0.026; proportion mediated: 30%] as well as between FTP SUVR and memory [total effect: *β* = −0.578, 95% CI (−1.089, −0.310), *P* < 0.001; direct effect: *β* = −0.421, 95% CI (−0.753, −0.180), *P* = 0.006; indirect effect: *β* = −0.157, 95% CI (−0.422, −0.010), *P* = 0.042; proportion mediated: 27%; Fig. 4C]. In the hippocampus, FDG SUVR was associated with memory beyond PiB DVR [total effect: *β* = −0.246, 95% CI (−0.637, 1.150), *P* = 0.580], while the association between FTP SUVR and memory was unaffected by FDG SUVR (mediator-outcome not significant; Fig. 4D).

## Discussion

Aβ and tau accumulation precede neurodegeneration in AD but their contribution to glucose hypometabolism and downstream cognition remains unclear. Thus, this multi-modal imaging study aimed to explore the local and global effects of Aβ and tau pathology on glucose metabolism as well as their interplay in memory decline in *PSEN1* E280A mutation carriers from the COLBOS Biomarker Study. We observed glucose metabolism reductions in parietal brain regions in *PSEN1* mutation carriers compared to non-carriers, which correlated with closer age to symptom onset. In carriers, hypometabolism correlated with greater tau accumulation in the medial temporal lobe, parietal and prefrontal regions, and with greater Aβ accumulation in the inferior temporal. These correlations were mainly explained by global pathology, except for frontal hypometabolism, which co-localized with tau accumulation independently of global tau pathology. Moreover, hypometabolism in the medial temporal lobe correlated with memory impairment in carriers. We found a region-specific interplay between AD biomarkers, glucose hypometabolism and memory performance, with hypometabolism in the inferior temporal mediating the effects of Aβ and tau burden on memory decline. Our study provides a comprehensive examination of the synergy between AD-specific pathology and hypometabolism and their impact on clinical symptoms that can help improve knowledge of the spatial and temporal progression of AD.

Glucose hypometabolism was most prominent in the precuneus and isthmus cingulate in *PSEN1* mutation carriers compared to non-carriers. In these brain regions, hypometabolism correlated with age in carriers and started to deviate from non-carriers approximately 15 and 12 years before the estimated age of MCI onset, respectively. This is in line with previous work carried out in ADAD showing precuneus hypometabolism 10–15 years prior to onset.^10-12,53^ Notably, in our Colombian kindred, cortical Aβ burden is elevated in *PSEN1* mutation carriers 16 years before MCI diagnosis.^11^ Beyond parietal regions, we also found a trend for hypometabolism in the superior temporal and middle frontal gyri in carriers compared to non-carriers, consistent with other ADAD studies.^26,53,54^ This pattern matches the well-known hypometabolism in typical sporadic AD in which parietal, temporal and frontal regions are progressively affected with disease severity.^3,5,7-9,55-57^ However, hemispheric asymmetries in *PSEN1* mutation carriers and normal aging should be further explored to obtain insights into disease-driven heterogeneities. Though early-onset AD patients present more severe hypometabolism in the precuneus than late-onset AD patients,^37^ our findings support a common pathophysiology between ADAD and sporadic AD that renders the precuneus metabolically vulnerable and demonstrate the value of ADAD to study the spatio-temporal disease trajectory.

Similarly, *PSEN1* mutation carriers displayed greater Aβ and tau accumulation than non-carriers, following typical patterns in ADAD.^26^  ^,52,58^ We observed regional correlations between greater tau burden and lower metabolism in the inferior temporal, entorhinal cortex, inferior parietal and isthmus cingulate. Previous studies have reported similar correlations in late-onset^23-25,59^ as well as early-onset AD.^18,26^ We further revealed that most correlations were no longer significant when controlled for global tau pathology. To our knowledge, our study is the first to account for local and global effects of pathology in ADAD. Though FDG-PET and FTP-PET are typically assumed to overlap and predict neuronal loss,^1^ our findings suggest a lack of local specificity between hypometabolism and tau accumulation in temporo-parietal brain regions, which is in agreement with the regional discrepancy between early tau deposition in the medial temporal lobe^52,60^ and early hypometabolism in the precuneus and posterior cingulate in AD. This discrepancy alludes to the idea of distant effects of tau pathology on hypometabolism, which originated from studies reporting tau-metabolism correlations in different but functionally connected brain regions.^23,24^

In contrast, we found a correlation between hypometabolism and tau accumulation in the caudal and rostral middle frontal regions, which was independent of global tau pathology. This suggests co-localization of tau tangles and deficient glucose utilization in the prefrontal cortex, a hub of the default mode network (DMN) that plays an important role in regulating complex cognition and behaviour.^61^ In this kindred, our group has shown that greater tau is linked with lower functional connectivity in the DMN, mainly in the precuneus and prefrontal cortex.^62^ Moreover, tau deposition, functional connectivity and metabolic covariance patterns have been shown to closely converge in sporadic AD.^63,64^ Despite higher Aβ pathology in the prefrontal cortex in *PSEN1* mutation carriers than non-carriers, the correlation between frontal tau accumulation and hypometabolism was mostly independent of Aβ accumulation, consistent with previous studies.^13,30,65^ Thus, frontal hypometabolism in our cohort could reflect dysconnectivity and neurodegeneration locally induced by the early spread of tau from the medial temporal lobe. More studies are critically needed to explore metabolic and functional networks in ADAD.

Despite cortex-wide Aβ accumulation in *PSEN1* mutation carriers, the regional association between Aβ burden and lower glucose metabolism was restricted to the inferior temporal gyrus in carriers. This correlation was mainly dependent on the global presence of Aβ, in line with observations in sporadic AD,^22^ indicating that inferior temporal hypometabolism does not result from regional deposits of fibrillary Aβ plaques. Some studies have observed local correlations between Aβ deposition and hypometabolism in temporo-parietal regions,^19-21^ whereas others found no correlations across the brain either in sporadic AD^15-17,59,65-67^ or ADAD.^18^ It has been proposed that long-term exposure to Aβ rather than absolute burden could lead to more severe hypometabolism or that the detrimental effect on metabolism is due to toxic Aβ oligomers instead of fibrillar Aβ as imaged by PiB-PET.^37^ This lack of correlation between Aβ and hypometabolism could also be interpreted as Aβ not playing a prominent role in ongoing neurodegeneration after reaching a plateau.

The link between reduced glucose metabolism and cognitive decline has been well described. Across the AD continuum, hypometabolism in the temporal lobe and posterior cingulate is associated with memory impairment, visuospatial and language deficits as well as global cognitive dysfunction.^6,29,31,32,35,68^ In our study, we also observed that lower glucose metabolism in the hippocampus, inferior temporal and entorhinal was associated with worse memory performance in *PSEN1* mutation carriers. Notably, these regions are involved in tau spatial spread, which highly correlates with cognition.^14,69^ Cognitive correlates of Aβ are, however, poorly understood,^33,67^ calling into question the contribution of AD hallmarks to memory deficits. We found that the interplay between Aβ, tau and glucose metabolism in memory performance was region-specific. In the precuneus and caudal middle frontal, greater Aβ and tau burden were associated with worse memory independently of hypometabolism. Similarly, in the hippocampus, the association between tau and memory deficits was not mediated by hypometabolism. This indicates that Aβ and tau pathology may affect cognition synergistically prior to neurodegeneration and supports the temporal ordering of events theorized in AD.^1^ Given the closer link between tau and hypometabolism in these regions, our findings also offer evidence for the role of tau as the primary driver of downstream pathology, as widely proposed in the literature.^27,70^

Interestingly, we found concurrent higher Aβ and tau accumulation, lower glucose metabolism and lower memory performance in the inferior temporal in *PSEN1* mutation carriers compared to non-carriers. The correlation between Aβ and hypometabolism was considerably reduced when controlling for local tau accumulation, in line with a study revealing that Aβ burden had an indirect effect on cerebral blood flow and volume in the inferior temporal gyrus via tau accumulation,^13^ considering that glucose hypometabolism mirrors both brain atrophy and brain hypoperfusion.^71,72^ However, the partial correlation between hypometabolism and tau controlling for Aβ accumulation, also remained significant, suggesting an intricate relationship between AD pathology and metabolic deficits in the inferior temporal. Both greater Aβ and tau burden were associated with worse memory, yet these effects were partially mediated (30%) by hypometabolism in this region. While mediation results must be interpreted with caution due to the non-causal exploratory nature of this statistical approach and the limited sample size, they suggest that these biomarkers might be independent contributors to cognitive decline in ADAD. These findings align with studies showing that hypometabolism may predict cognitive decline independently of tau in sporadic AD and ADAD.^34,35,73^ The potential FDG-PET’s ability to independently predict cognitive decline might be explained by hypometabolism being closer in time to symptom onset compared to Aβ and tau accumulation. We could hypothesize that the mechanisms through which inefficient glucose utilization in certain vulnerable brain regions leads to neurodegeneration may occur regardless of the presence of AD pathology,^74^ which would have important implications in disease management. Ultimately, our findings contribute to the growing evidence that supports the introduction of combination therapy aiming to slow down cognitive decline due to tau pathology as well as deficient glucose utilization. Overall, the present study describes a model where pathology advances and interacts in a region-specific manner to impact clinical outcomes, underscoring the importance of differential regional vulnerability in AD, with FDG-PET demonstrating to be an invaluable biomarker to unveil the spatial progression of pathology. More studies of how regional glucose hypometabolism relates to the wider spectrum of cognitive domains are warranted to provide a more comprehensive understanding of regional vulnerability expression and clinical interpretation of FDG-PET.

Although the examination of a single ADAD mutation provides methodological and conceptual benefits, this study has multiple limitations. Our Colombian kindred has a high load of Aβ pathology but not tau pathology compared to sporadic AD,^58^ which limits the generalizability of our findings. In addition, the small sample size precludes us from building more complex mediation models to further investigate the intercorrelations between the different biomarkers. Other methodological limitations include the lack of medication data among carriers and the reference region selection, which may be prone to off-target binding of FTP.^75^ Moreover, the cross-sectional nature of this study hinders causal interpretations of our mediation findings. To that end, longitudinal studies are needed to clarify the interactions between progression of Aβ, tau and glucose hypometabolism across the AD spectrum. Finally, FDG-PET is an unspecific marker of brain health that reflects not only neurodegeneration, but vascular dysfunction as well, which was not accounted for in this study.^71,72,76^ Our study also has multiple strengths. This unique cohort of *PSEN1* mutation carriers with multi-modal imaging available has a deterministic trajectory of pathological and clinical decline. Given that the mean age of symptom onset is 44 years old,^36^ we are able to examine different biomarkers without the confound of normal aging. Additionally, due to the young age of this cohort, the prevalence of other potential confounders such as vascular risk is relatively low.

## Supplementary Material

fcaf508_Supplementary_Data

## Data Availability

Anonymized clinical, genetic and imaging data are available upon request, subject to an internal review by Y.T.Q. to ensure that the participants’ anonymity, confidentiality, and *PSEN1* E280A carrier or non-carrier status are protected. Data requests will be considered based on a proposal review, and completion of a data sharing agreement, in accordance with the University of Antioquia and Mass General Brigham institutional guidelines. Please submit data requests to Y.T.Q.
