# Supplementary material for: Contribution of local amyloid-β and tau burden to hypometabolism in autosomal-dominant Alzheimer’s disease
Source: Brain Commun. 2026 Jan 2;8(1):fcaf508. doi: 10.1093/braincomms/fcaf508 (PMC12816921; doi:10.1093/braincomms/fcaf508)
Supplement: fcaf508_Supplementary_Data [file fcaf508_supplementary_data.docx]

**Contribution of local amyloid-β and tau burden to hypometabolism in autosomal-dominant Alzheimer’s disease**

**Running title**: Local Aβ, tau and hypometabolism in ADAD

Catarina Tristão-Pereira, Stephanie Langella, Ana Baena, Natalia Londono, Justin S. Sanchez, Lusiana Martinez, Sergio Alvarez, Monica Vidal, David Aguillon, Yi Su, Hillary Protas, Michael John Properzi, Vincent Malotaux, Bing He, Averi Giudicessi, Eric Reiman, Bernard J. Hanseeuw and Yakeel T. Quiroz

**Supplementary Material**

**Supplementary Methods.** Neuropsychological assessments......................................................... 2

**Supplementary Table 1.** Demographics of the imaging sub-sample............................................. 3

**Supplementary Table 2.** Adjusted regional FDG SUVR correlations in *PSEN1* mutation carriers............................................................................................................................................. 4

**Supplementary Table 3.** Local and global effects of Aβ and tau accumulation on glucose metabolism in the whole sample...................................................................................................... 5

**Supplementary Figure 1.** Regional glucose metabolism differences between left and right hemispheres across ADAD.............................................................................................................. 6

**Supplementary Figure 2.** Regional Aβ and tau accumulation differences between *PSEN1* mutation carriers and non-carriers................................................................................................... 7

**Supplementary Figure 3.** Regional association between glucose metabolism and Aβ and tau pathology in non-carriers................................................................................................................. 8

**Supplementary Figure 4.** Association between regional glucose metabolism and memory performance in non-carriers............................................................................................................. 9

**Supplementary Figure 5.** Association between regional glucose metabolism and semantic fluency and processing speed in *PSEN1* mutation carriers........................................................... 10

**References**.................................................................................................................................... 11

**Supplementary Methods. Neuropsychological assessments.**

Neuropsychological assessments included the Consortium to Establish a Registry for Alzheimer’s Disease (CERAD) Word List Memory task, which is divided into learning, free recall and recognition. In particular, the word list learning is the score calculated as the sum of words recalled correctly over 3 trials after participants are shown 10 cards for 2 seconds each. This battery has been validated to distinguish between Alzheimer’s disease (AD) and cognitively normal aging^1^ and the Spanish CERAD version has been standardized in Colombia.^2^ Beyond memory, the CERAD protocol also includes a semantic fluency test and the modified Boston naming test. In semantic fluency, participants are asked to generate as many words as possible that fit a particular category (e.g., animals) within 1 minute.^3^ Among assessments of executive functioning, participants underwent the Trail Making Test Part A, in which they are asked to connect numbered circles in ascending order as quickly as possible. The time to complete the task measures processing speed.^4,5^ A comprehensive list of neuropsychological assessments available in the Colombian ADAD kindred has been detailed elsewhere.^6,7^

**Supplementary Table 1. Demographics of the imaging sub-sample.** Continuous variables are represented as mean ± standard deviation and categorical variables as n (%). Demographics were compared between *PSEN1* mutation carriers (n=22) and non-carriers (n=26) with available PiB-PET and FTP-PET data using two-tailed t-tests for continuous variables and χ^2^-tests for categorical variables. Bold font represents statistical significance (p < 0.05). CERAD = Consortium to Establish a Registry for Alzheimer’s Disease**,** DVR = distribution value ratio, FAST = Functional Assessment Staging Tool, FTP = [18F]flortaucipir, MMSE = Mini Mental State Examination, PiB = [11C]Pittsburgh compound B, SUVR = standardized uptake value ratio, TMTA = Trail Making Test Part A. ^1^Differences between groups within the imaging sub-sample; ^2^Differences between the imaging sub-sample and the remaining sample.

|  | ***PSEN1* E280A carriers**  **(n=22)** | **Non-**  **carriers**  **(n=26)** | **p-value^1^** | **p-value^2^** |
| --- | --- | --- | --- | --- |
| **Age (years)** | 42.0 ± 6.7 | 38.5 ± 5.7 | 0.062 | 0.078 |
| **Education (years)** | 8.5 ± 4.3 | 11.7 ± 4.1 | 0.140 | 0.130 |
| **Sex (n, %)** |  |  | 0.639 | 0.662 |
| Female | 12 (55%) | 17 (65%) |  |  |
| Male | 10 (45%) | 15 (35%) |  |  |
| **FAST** |  |  | **<0.001** | 0.066 |
| ≤ 2 | 16 (73%) | 26 (100%) |  |  |
| > 2 | 6 (27%) | 0 |  |  |
| **MMSE** | 26.7 ± 3.0 | 29.1 ± 0.8 | **0.001** | 0.886 |
| **CERAD’s word list learning** | 15.5 ± 5.2 | 21.6 ± 3.1 | **<0.001** | 0.206 |
| **CERAD’s semantic fluency** | 20.7 ± 5.2 | 21.0 ± 3.4 | **0.835** | 0.341 |
| **TMTA time** | 66.5 ± 48.2 | 38.9 ± 13.9 | **0.016** | 0.064 |
| **PiB DVR (global Aβ burden)** | 1.71 ± 0.40 | 1.12 ± 0.05 | **<0.001** | - |
| **FTP SUVR (global tau burden)** | 1.77 ± 0.80 | 1.10 ± 0.13 | **<0.001** | - |

**Supplementary Table 2. Adjusted regional FDG SUVR correlations in *PSEN1* mutation carriers.** FDG SUVR correlations with age, FTP SUVR, PiB DVR and memory performance (CERAD’s word list learning) in *PSEN1* E280A carriers adjusted for MMSE (global cognition) using partial Spearman correlation. Spearman correlation coefficients *ρ*, bootstrap-derived 95% confidence intervals (CI) and p-values are shown for each correlation. Bold font represents statistical significance (p < 0.05). CERAD = Consortium to Establish a Registry for Alzheimer’s Disease, DVR = distribution value ratio, FDG = [18F]fluorodeoxyglucose, FTP = [18F]flortaucipir, PiB = [11C]Pittsburgh compound B, SUVR = standardized uptake value ratio, WLL = word list learning.

| **Correlation** | **MMSE-adjusted partial correlations** | |
| --- | --- | --- |
|  | ***ρ* [95% CI]** | **p-value** |
| **Age (n=43):** |  |  |
| Precuneus FDG SUVR | -0.531 [-0.717, -0.274] | **<0.001** |
| Isthmus cingulate FDG SUVR | -0.581 [-0.750, -0.340] | **<0.001** |
| **Same-region FTP SUVR (n=22):** |  |  |
| Caudal middle frontal FDG SUVR | -0.841 [-0.932, -0.650] | **<0.001** |
| Inferior temporal FDG SUVR | -0.715 [-0.873, -0.420] | **<0.001** |
| **Same-region PiB DVR (n=22):** |  |  |
| Inferior temporal FDG SUVR | -0.704 [-0.868, -0.402] | **<0.001** |
| Inferior parietal FDG SUVR | -0.512 [-0.768, -0.115] | **0.018** |
| **CERAD WLL (n=43):** |  |  |
| Hippocampus FDG SUVR | 0.465 [0.191, 0.671] | **0.002** |
| Inferior temporal FDG SUVR | 0.398 [0.111, 0.624] | **0.009** |

**Supplementary Table 3. Local and global effects of Aβ and tau accumulation on glucose metabolism in the whole sample.** Local simple and partial correlations of FDG SUVR with FTP SUVR and PiB DVR in the imaging sub-sample (n=48) in brain regions where simple correlations survived Bonferroni correction. Partial correlations were either controlled for global FTP SUVR or same-region PiB DVR and for global PiB DVR or same-region FTP SUVR. Spearman correlation coefficients *ρ*, bootstrap-derived 95% confidence intervals (CI) and p-values are shown for each correlation. Bold font represents statistical significance (p < 0.05). DVR = distribution value ratio, FDG = [18F]fluorodeoxyglucose, FTP = [18F]flortaucipir, PiB = [11C]Pittsburgh compound B, SUVR = standardized uptake value ratio.

| **Partial correlations between local FDG SUVR and FTP SUVR in the whole sample** | | | | |
| --- | --- | --- | --- | --- |
| **Region** | **Simple**  **correlation** | **Controlling for**  **global FTP SUVR** | **Controlling for**  **local PiB DVR** |  |
| **Caudal Middle Frontal** | **-0.503**  **[-0.716, -0.225]**  **(p < 0.001)** | **-0.377**  **[-0.597, -0.104]**  **(p = 0.009)** | **-0.458**  **[-0.657, -0.200]**  **(p = 0.001)** |  |
| **Inferior Temporal** | **-0.473**  **[-0.681, -0.209]**  **(p < 0.001)** | -0.267  [-0.512, 0.019]  (p = 0.070) | **-0.352**  **[-0.578, -0.075]**  **(p = 0.015)** |  |
| **Entorhinal** | -0.239  [-0.507, 0.089]  (p = 0.101) | -0.139  [-0.407, 0.151]  (p = 0.350) | **-0.415**  **[-0.626, -0.149]**  **(p = 0.004)** |  |
| **Inferior Parietal** | **-0.452**  **[-0.678, -0.161]**  **(p = 0.001)** | -0.223  [-0.477, 0.066]  (p = 0.132) | **-0.372**  **[-0.594, -0.099]**  **(p = 0.010)** |  |
| **Rostral Middle Frontal** | **-0.501**  **[-0.701, -0.233]**  **(p < 0.001)** | **-0.312**  **[-0.547, -0.030]**  **(p = 0.033)** | **-0.400**  **[-0.615, -0.131]**  **(p = 0.005)** |  |
| **Isthmus Cingulate** | **-0.475**  **[-0.696, -0.161]**  **(p < 0.001)** | -0.181  [-0.443, 0.108]  (p = 0.222) | **-0.357**  **[-0.582, -0.081]**  **(p = 0.014)** |  |
| **Partial correlations between local FDG SUVR and PiB DVR in the whole sample** | | | | |
| **Region** | **Simple**  **correlation** | **Controlling for**  **global PiB DVR** | **Controlling for**  **local FTP SUVR** |  |
| **Inferior Temporal** | **-0.353**  **[-0.600, -0.029]**  **(p = 0.014)** | -0.188  [-0.565, 0.254]  (p = 0.414) | -0.109  [-0.381, 0.181]  (p = 0.467) |  |

**Supplementary Figure 1.** **Regional glucose metabolism differences between left and right hemispheres across ADAD.** Ordered effect sizes of the left and right differences in regional FDG SUVR in *PSEN1* E280A carriers (n=43) (**A**) and non-carriers (n=39) (**B**), calculated as the rank-biserial correlation r of the paired Wilcoxon rank-sum test (dots). Whiskers represent upper and lower bounds of bootstrap-derived 95% confidence intervals. Brain projections highlight the regions with statistically significant differences between groups (*p* < 0.05), with negative effect sizes (light blue) indicating lower FDG SUVR in the left hemisphere and positive effect sizes (light red) indicating lower FDG SUVR in the right hemisphere. Regions highlighted within the forest plot represent models that survived Bonferroni correction for multiple comparisons. *: *p*_adj_ < 0.05, **: *p*_adj_ < 0.01, ***: *p*_adj_ < 0.001. FDG = [18F]fluorodeoxyglucose, SUVR = standardized uptake value ratio, Banks Sup Temp Sulcus = banks of the superior temporal sulcus, Ventral DC = ventral diencephalon.


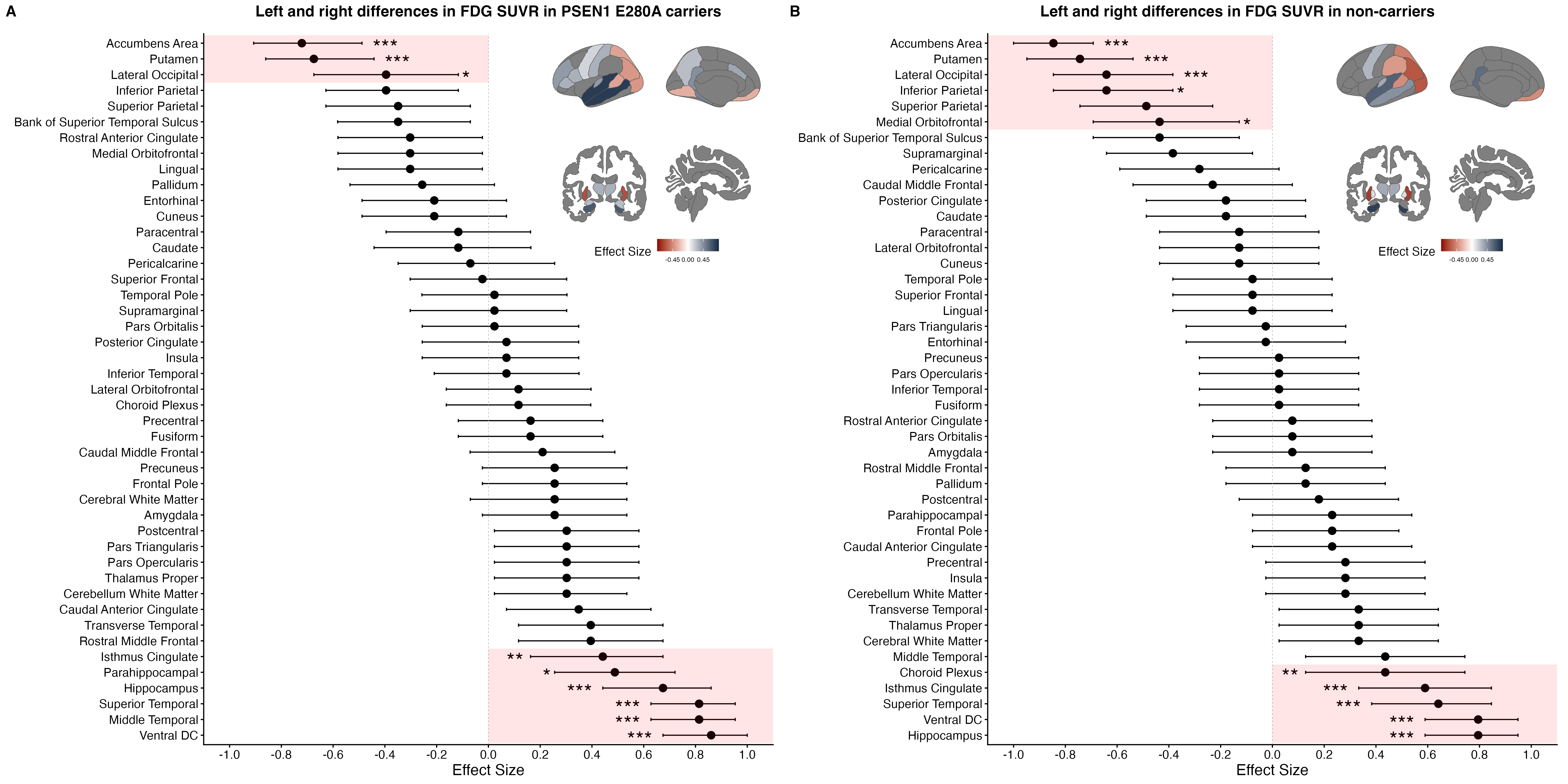


**Supplementary Figure 2.** **Regional Aβ and tau accumulation differences between *PSEN1* mutation carriers and non-carriers.** Ordered effect sizes of the differences in regional FTP SUVR (**A**) and PiB DVR (**B**) between groups calculated as the rank-biserial correlation r of the Wilcoxon rank-sum test (dots, n=48). Whiskers represent upper and lower bounds of bootstrap-derived 95% confidence intervals. Positive effect sizes indicate higher FTP SUVR and PiB DVR in *PSEN1* mutation carriers compared to non-carriers. Brain projections highlight the regions with statistically significant differences between groups (*p* < 0.05), with light blue representing larger effect sizes. Regions highlighted within the forest plot represent models that survived Bonferroni correction for multiple comparisons. *: *p*_adj_ < 0.05, **: *p*_adj_ < 0.01, ***: *p*_adj_ < 0.001. DVR = distribution value ratio, FTP = [18F]flortaucipir, PiB = [11C]Pittsburgh compound B, SUVR = standardized uptake value ratio, Banks Sup Temp Sulcus = banks of the superior temporal sulcus, Ventral DC = ventral diencephalon.


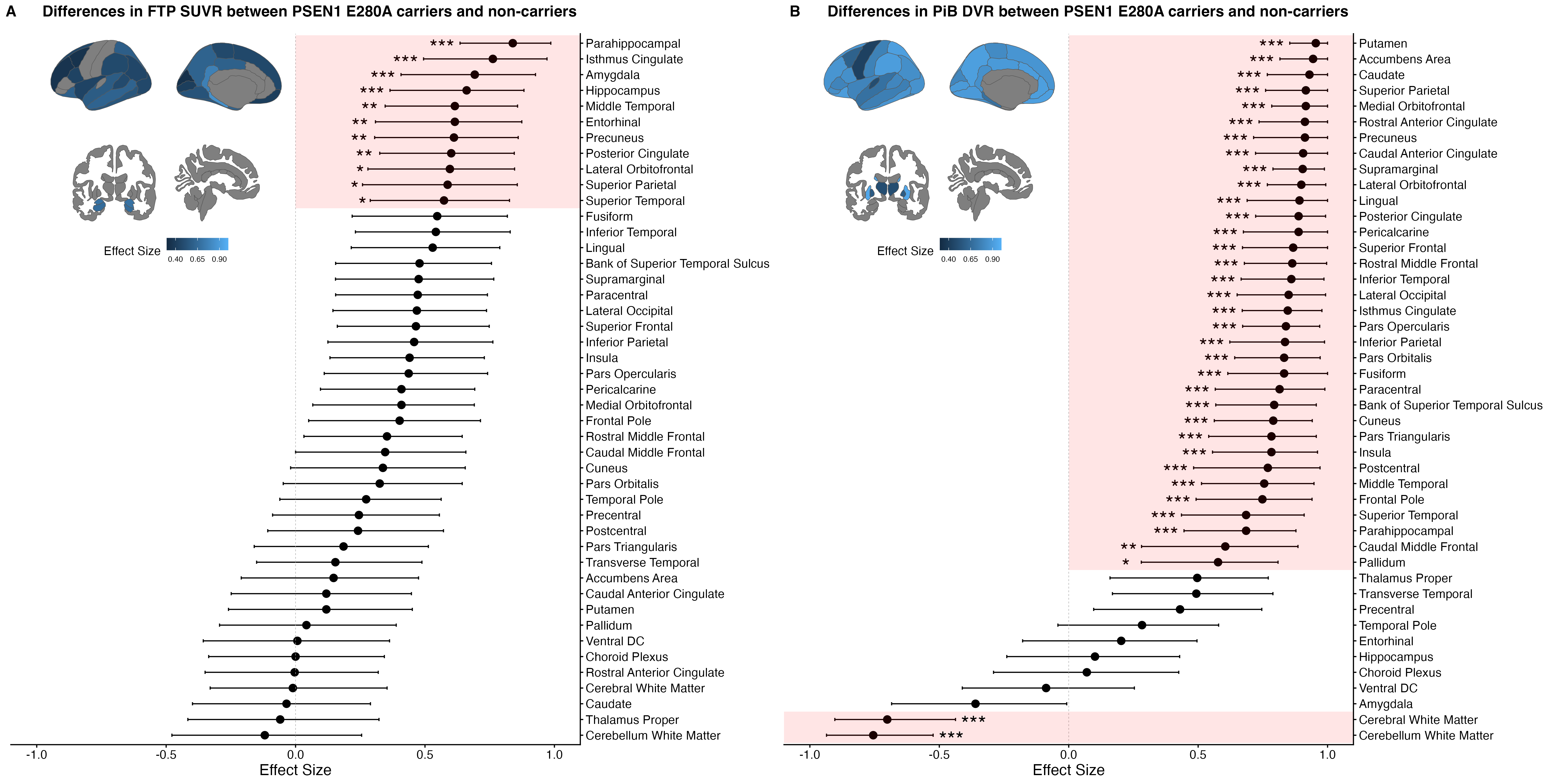


**Supplementary Figure 3. Regional association between glucose metabolism and Aβ and tau pathology in non-carriers.** Ordered correlation coefficients *ρ* of the regional associations of FDG SUVR with FTP SUVR (**A**) and PiB DVR (**B**) in non-carriers (dots, n=26) calculated from Spearman correlation. Whiskers represent upper and lower bounds of bootstrap-derived 95% confidence intervals. Brain projections highlight the regions with statistically significant correlations, with light blue representing larger effect sizes for positive correlations. No regions survived Bonferroni correction for multiple comparisons. DVR = distribution value ratio, FDG = [18F]fluorodeoxyglucose, FTP = [18F]flortaucipir, PiB = [11C]Pittsburgh compound B, SUVR = standardized uptake value ratio, Banks Sup Temp Sulcus = banks of the superior temporal sulcus, Ventral DC = ventral diencephalon.

**
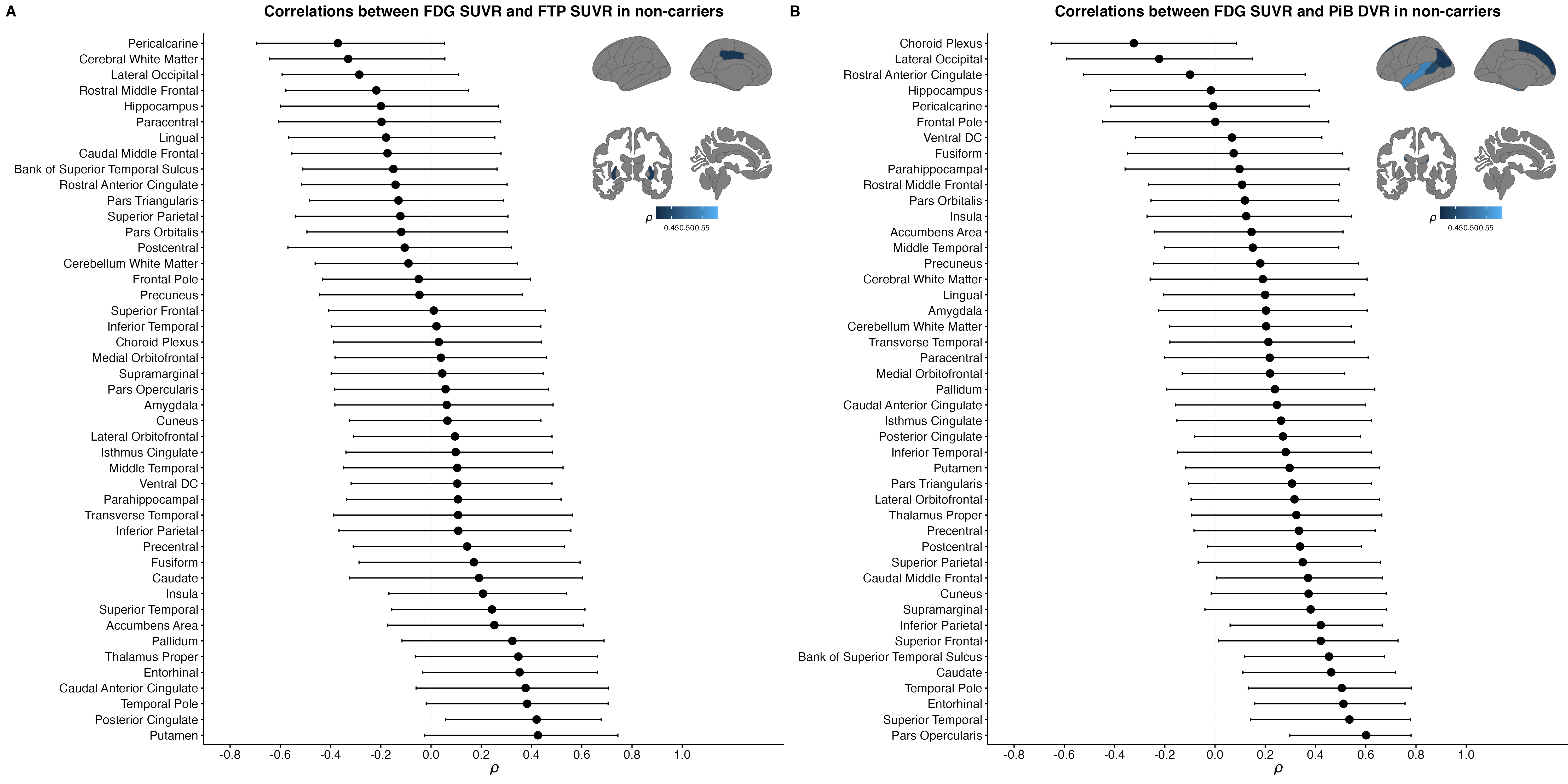
**

**Supplementary Figure 4. Association between regional glucose metabolism and memory performance in non-carriers.** Ordered correlation coefficients *ρ* of the associations of regional FDG SUVR with memory performance (CERAD word list learning) in non-carriers (dots, n=39) calculated from Spearman correlation. Whiskers represent upper and lower bounds of bootstrap-derived 95% confidence intervals. Brain projections highlight the regions with statistically significant correlations, with light red representing larger effect sizes for negative correlations. No regions survived Bonferroni correction for multiple comparisons. FDG = [18F]fluorodeoxyglucose, SUVR = standardized uptake value ratio, Banks Sup Temp Sulcus = banks of the superior temporal sulcus, Ventral DC = ventral diencephalon.


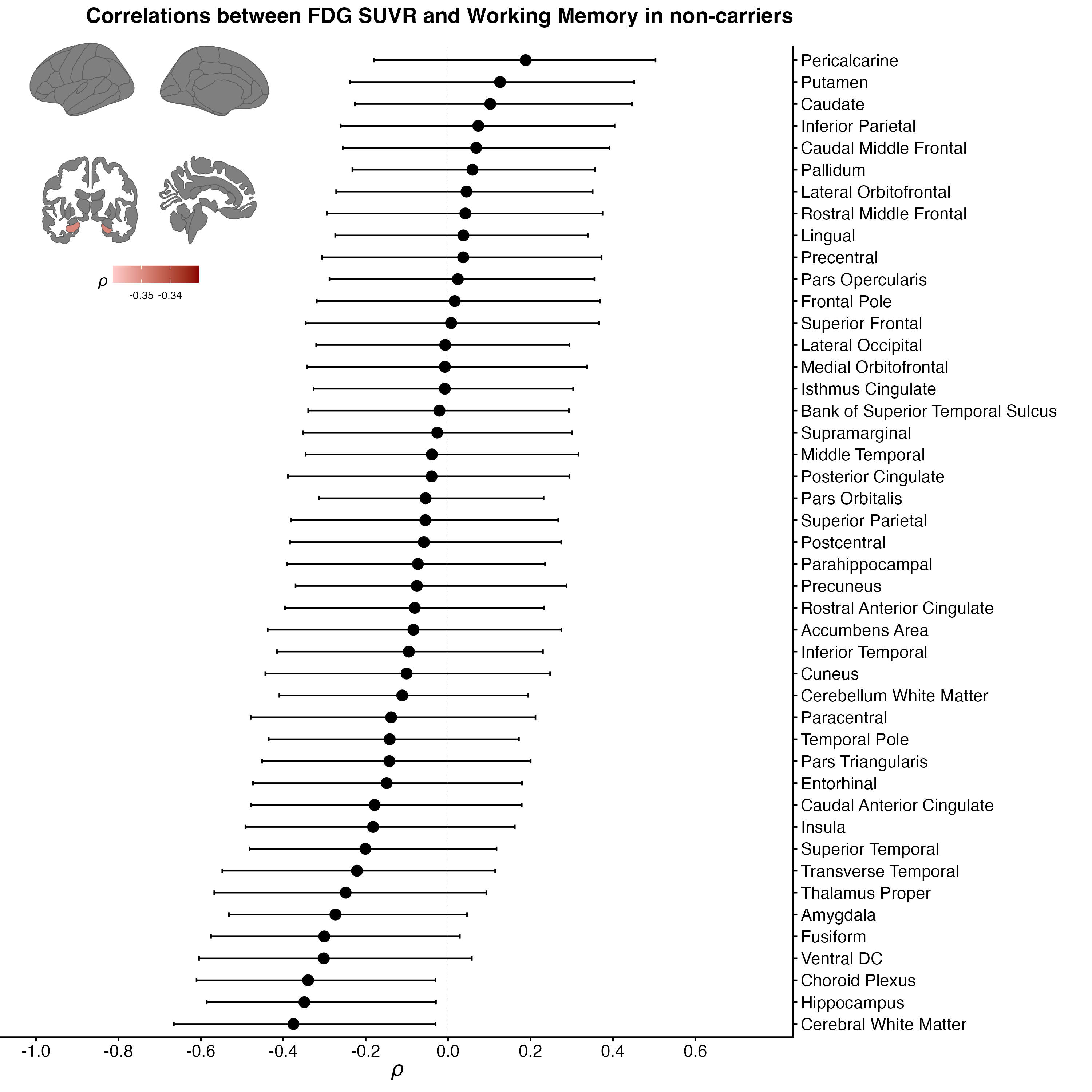


**Supplementary Figure 5.** **Association between regional glucose metabolism and semantic fluency and processing speed in *PSEN1* mutation** **carriers.** Ordered correlation coefficients *ρ* of the associations of regional FDG SUVR with semantic fluency (CERAD’s semantic fluency test, **A**) and processing speed (TMTA time, **B**) in *PSEN1* mutation carriers (dots, n=43) calculated from Spearman correlation. Whiskers represent upper and lower bounds of bootstrap-derived 95% confidence intervals. Brain projections highlight the regions with statistically significant correlations, with light red representing larger effect sizes for negative correlations. Regions highlighted within the forest plot represent models that survived Bonferroni correction for multiple comparisons. *: *p*_adj_ < 0.05. CERAD = Consortium to Establish a Registry for Alzheimer’s disease, FDG = [18F]fluorodeoxyglucose, SUVR = standardized uptake value ratio, TMTA = Trail Making Test Part A, Banks Sup Temp Sulcus = banks of the superior temporal sulcus, Ventral DC = ventral diencephalon.


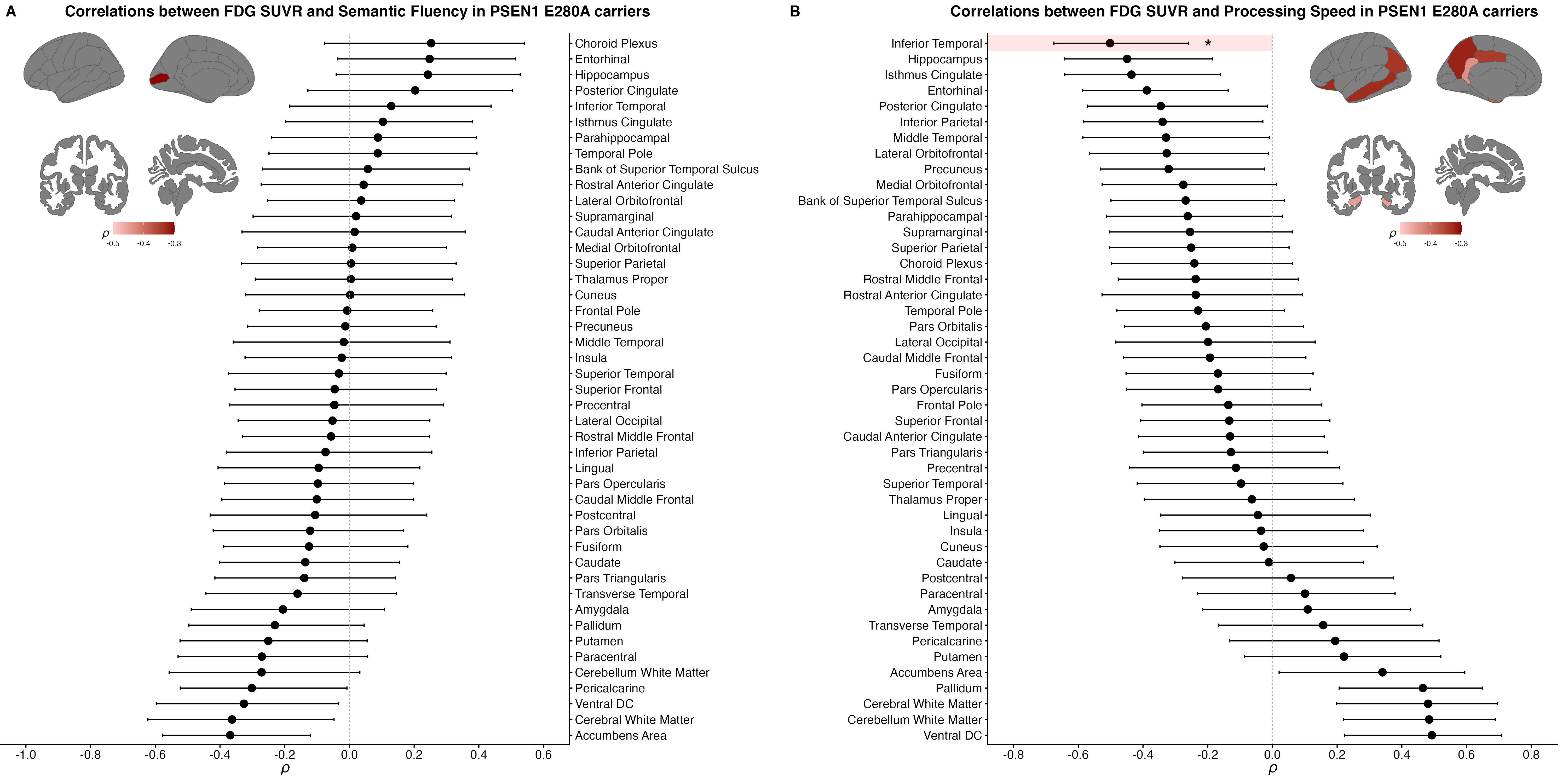


**References**

1. Welsh KA, Butters N, Mohs RC, et al. The Consortium to Establish a Registry for Alzheimer’s Disease (CERAD). Part V. A normative study of the neuropsychological battery. *Neurology*. 1994;44(4):609-609. doi:10.1212/WNL.44.4.609

2. Aguirre-Acevedo DC, Gómez RD, Moreno S, et al. [Validity and reliability of the CERAD-Col neuropsychological battery]. *Rev Neurol*. 2007;45(11):655-660.

3. Strauss E, Sherman EMS, Spreen O, Spreen O. *A Compendium of Neuropsychological Tests: Administration, Norms, and Commentary*. 3rd ed. Oxford University Press; 2006.

4. Reitan RM. Validity of the Trail Making Test as an Indicator of Organic Brain Damage. *Percept Mot Skills*. 1958;8(3):271-276. doi:10.2466/pms.1958.8.3.271

5. Llinàs-Reglà J, Vilalta-Franch J, López-Pousa S, Calvó-Perxas L, Torrents Rodas D, Garre-Olmo J. The Trail Making Test. *Assessment*. 2017;24(2):183-196. doi:10.1177/1073191115602552

6. Giudicessi A, McDowell CP, Martinez JE, et al. Cognitive Outcomes in Autosomal-Dominant Alzheimer’s Disease: A Comprehensive Review from a Colombian Kindred with the Presenilin-1 E280A Mutation. Alegret M, ed. *JAD*. 2024;101(2):397-415. doi:10.3233/JAD-240360

7. Acosta-Baena N, Sepulveda-Falla D, Lopera-Gómez CM, et al. Pre-dementia clinical stages in presenilin 1 E280A familial early-onset Alzheimer’s disease: a retrospective cohort study. *The Lancet Neurology*. 2011;10(3):213-220. doi:10.1016/S1474-4422(10)70323-9
